# Accumulation and Competition Amongst Deformed Wing Virus Genotypes in Naïve Australian Honeybees Provides Insight Into the Increasing Global Prevalence of Genotype B

**DOI:** 10.3389/fmicb.2020.00620

**Published:** 2020-04-09

**Authors:** Amanda M. Norton, Emily J. Remnant, Gabriele Buchmann, Madeleine Beekman

**Affiliations:** Behaviour, Ecology and Evolution (BEE) Laboratory, School of Life and Environmental Sciences, The University of Sydney, Sydney, NSW, Australia

**Keywords:** RNA viruses, accumulation, competition, virulence, honeybee, parasites, pathogens, *Varroa*

## Abstract

Honeybee colony deaths are often attributed to the ectoparasitic mite *Varroa destructor* and deformed wing virus (DWV), vectored by the mite. In the presence of *V. destructor* both main genotypes (DWV-A and DWV-B) have been correlated with colony loss. Studies show that DWV-B is the most prevalent genotype in the United Kingdom and Europe. More recently DWV-B has increased in prevalence in the United States. The increasing prevalence of DWV-B at the expense of DWV-A suggests that competition exists between the genotypes. Competition may be due to disparities in virulence between genotypes, differences in fitness, such as rate of replication, or a combination of factors. In this study we investigated if DWV genotypes differ in their rate of accumulation in Australian honeybees naïve to both *V. destructor* and DWV, and if viral load was associated with mortality in honeybee pupae. We singly and co-infected pupae with DWV-A, DWV-B, and a recombinant strain isolated from a *V. destructor* tolerant bee population. We monitored viral accumulation throughout pupation, up to 192 h post-injection. We found significant differences in accumulation, where DWV-A accumulated to significantly lower loads than DWV-B and the DWV-recombinant. We also found evidence of competition, where DWV-B loads were significantly reduced in the presence of DWV-A, but still accumulated to the highest loads overall. In contrast to previous studies, we found significant differences in virulence between pupae injected with DWV-A and DWV-B. The average mortality associated with DWV-B (0.4% ± 0.33 SE) and DWV-recombinant (2.2% ± 0.83 SE) injection were significantly less than observed for DWV-A (11% ± 1.2 SE). Our results suggest that a higher proportion of DWV-B infected pupae will emerge into adults, compared to DWV-A. Overall, our data suggest that low mortality in pupae and the ability of DWV-B to accumulate to higher loads relative to DWV-A even during co-infection may favor vector transmission by *V. destructor*, and may thus be contributing factors to the increasing prevalence of DWV-B globally.

## Introduction

*Varroa destructor* is arguably one of the biggest threats to Western honeybee (*Apis mellifera*) populations worldwide. Over the past 60 years, *V*. *destructor* has spread globally from its origin in Asia where the mite originally parasitized the Asian honeybee *Apis cerana* (Solignac et al., 2005). *V*. *destructor* parasitism is particularly destructive to *A. mellifera* (hereafter simply honeybees), and is associated with significant colony losses. Australia is currently the only major beekeeping country to remain free from *V*. *destructor* (Oldroyd, 1999; Roberts et al., 2017).

Honeybee colony losses associated with *V*. *destructor* have often been attributed to viruses vectored by mites during feeding. One virus in particular, deformed wing virus (DWV), is frequently associated with *V. destructor* (Highfield et al., 2009; Martin et al., 2012; Mondet et al., 2014; Martin and Brettell, 2019). DWV is a single-stranded positive sense RNA virus belonging to the *Iflaviridae* family. Prior to the spread of *V. destructor*, DWV was rarely detected whereas now the virus is found in virtually all honeybee populations worldwide, excluding Australia (Roberts et al., 2017). In some *Varroa*-free honeybee populations, DWV has been shown to have low prevalence and accumulate to very low levels (Martin et al., 2012; Ryabov et al., 2014; Shutler et al., 2014; McMahon et al., 2016). In contrast, numerous studies have found a positive correlation between *V. destructor* infestation levels and increased DWV loads (Martin et al., 2012; Nazzi et al., 2012; Mondet et al., 2014; Wu et al., 2017). Within a *V. destructor* infested colony, vector transmission of DWV is associated with approximately 20% pupal mortality (Martin, 2001; Martin et al., 2013). Such relatively low mortality allows the majority of DWV infected brood to emerge as adults. And because *V. destructor* reproduces within honeybee brood cells (Martin, 1995), low brood mortality results in a continuing increase in the number of mites and transmission of DWV.

Three DWV genotypes have been described: DWV-A, DWV-B, and DWV-C (Mordecai et al., 2016b); only DWV-A (formally DWV) and DWV-B [formally *V. destructor virus* 1 or VDV-1 (Ongus et al., 2004)] are currently recognized by the International Committee on Taxonomy of Viruses. For clarity, we have employed the types A and B nomenclature widely adopted in recent publications (Martin et al., 2012; McMahon et al., 2016; Mordecai et al., 2016a, b; Brettell and Martin, 2017; Kevill et al., 2017; Gisder et al., 2018; Brettell et al., 2019; Dubois et al., 2019; Kevill et al., 2019; Remnant et al., 2019; Tehel et al., 2019).

An immediate effect of *V. destructor* appears to be a reduction in the genetic diversity of DWV in honeybees both in the field (Martin et al., 2012) and in experiments using injection of DWV to mimic vector transmission (Ryabov et al., 2014). Over time, the distribution of DWV genotypes changes so that one DWV genotype prevails within honeybee populations. DWV-B has become the most common variant in the United Kingdom (UK) and Europe (McMahon et al., 2016; Kevill et al., 2019; Manley et al., 2019). In North-America DWV-A remains the most common genotype (Ryabov et al., 2017; Kevill et al., 2019). However, Ryabov et al. (2017) found that DWV-B prevalence in the United States increased from 3% in 2010 to 65% in 2016. Similarly, Kevill et al. (2019) found that DWV-B was prevalent in 56% of tested colonies in 2016, and the dominant genotype in 23% of those colonies. Kevill et al. (2019) predicted that DWV-B prevalence will continue to increase and supersede DWV-A with time, as observed in England and Wales. Such change in relative prevalence suggests that the different DWV genotypes compete within their host. The increased prevalence of DWV-B may potentially be explained by differences in replication rate within the host, difference in virulence and associated host mortality, or a combination of both.

Understanding the exact relationship between DWV genotype and host virulence is far from straightforward. Not all studies distinguish between DWV genotypes. In those studies that do both DWV-A (Highfield et al., 2009; Martin et al., 2012; Mondet et al., 2014; Kevill et al., 2017, 2019; Barroso-Arévalo et al., 2019b) and DWV-B (Natsopoulou et al., 2017) have been associated with colony deaths in the presence of *V. destructor*. At the same time, high viral loads of DWV-B have been associated with low levels of colony mortality in the United Kingdom and Spain (Mordecai et al., 2016a; Barroso-Arévalo et al., 2019b; Kevill et al., 2019). High DWV-B loads in surviving colonies that were untreated for *V. destructor* led Mordecai et al. (2016a) to hypothesize that DWV-B may outcompete DWV-A via ‘superinfection exclusion.’ If DWV-B is a superior competitor, it could prevent DWV-A from replicating to high levels. If, then, DWV-B causes less damage to the host, the exclusion of the more harmful DWV-A genotype could result in the association between DWV-B and low honeybee mortality.

Despite evidence of colonies surviving with high DWV-B loads, experimental evidence thus far indicates that DWV-B is more harmful to adult honeybees than DWV-A. After DWV-B was injected into adult bees, the virus was detected in brain tissue which was associated with impairment of cognitive function (Gisder et al., 2018). The inocula were then serially passaged in pupae before a second round of adult injections. After one round of serial passage viral particles were not detected in the bees’ brain and the bees did not suffer from cognitive impairment. Gisder et al. (2018) associated the decreased tissue tropism and virulence of the passaged inoculum with a concurrent sequence shift from DWV-B to DWV-A. In a different study, injection of DWV-B into newly emerged adults resulted in significantly altered foraging behavior and higher mortality compared to controls (Benaets et al., 2017). However, the same experiment was not conducted on DWV-A (Benaets et al., 2017). When DWV-A and DWV-B were compared in a separate study, injection of DWV-B into newly emerged adults resulted in a significant reduction in survival compared to DWV-A (McMahon et al., 2016).

The most likely life stage to be infected with DWV is the pupal stage. Models have suggested that vector transmission of DWV to pupae results in reduced longevity in emerging adult honeybees and can lead to colony death in temperate climates, due to significantly reduced overwinter workforce (Martin, 2001; Sumpter and Martin, 2004). Thus, some studies have assessed whether DWV genotypes affect pupae differently. Gisder et al. (2018) found that injection of DWV-B into pupae resulted in significantly higher mortality compared to pupae injected with the passaged inoculum. Lamp et al. (2016) did not test DWV-B, but showed that both DWV-A directly isolated from infected bees and a constructed molecular clone both caused pupal death. However, Tehel et al. (2019) found no difference in survival between pupae injected with DWV-A or DWV-B when pupae were injected with the same source inocula as McMahon et al. (2016). Dubois et al. (2019) also found no difference in mortality between pupae infected with DWV-A and DWV-B obtained from heads of naturally infected bees. Similarly, a study using *V*. *destructor* and DWV naïve Australian honeybee pupae found no significant pupal mortality when white-eyed pupae were injected with DWV-A isolated from adult bees with overt disease symptoms, including deformed wings (Remnant et al., 2019).

Clearly while the global association between *V. destructor* and DWV seems irrefutable, determining whether virulence differences exist between DWV genotypes remains a challenge. The aforementioned experimental studies differ in many attributes, such as source inocula [with the exception of (McMahon et al., 2016; Tehel et al., 2019)], bee populations, life stage infected, experiment duration, and potential presence of other pathogens. In addition, covert infections with DWV may affect results as injection with buffered salt solutions can activate DWV replication (Dubois et al., 2019; Posada-Florez et al., 2019; Tehel et al., 2019). Similarly, previous infestations with *V. destructor* may have changed the viral landscape within honeybee populations by selecting for particular DWV genotypes that are better adapted to vector-based transmission. Australian honeybees are naïve to both *V. destructor* and DWV and are therefore an ideal model to determine the dynamics between different DWV genotypes. We infected white-eyed pupae to reflect the life stage at which *V. destructor* first vectors DWV to honeybees (Bailey and Ball, 1991). We infected pupae by injecting either a single DWV genotype or two genotypes (co-infection). Co-infection allowed us to determine the extent to which different DWV genotypes compete within the same host. We further determined if there is a relationship between viral load and host damage (mortality).

## Materials and Methods

### DWV Source Material and Strain Confirmation

Inocula were prepared from individual asymptomatic adult bees collected from Blenheim, New Zealand (DWV-A) and Amsterdam Water Dunes, the Netherlands (DWV-B and DWV-recombinant). The New Zealand bees were collected from *V. destructor* treated colonies and the Netherlands bees were collected from colonies that were part of a selection program for *V. destructor* tolerance (Panziera et al., 2017). The bees were imported on dry ice and stored at −80°C (Import permit and Quarantine details below). As we intended to use the source material as inocula, we firstly extracted viral material from individual bees from each population [protocol adapted from Remnant et al. (2019)]. We homogenized the thorax and abdomen [as eye pigments have been shown to inhibit PCR reactions (Boncristiani et al., 2011)] of individual adults in 2 mL 0.5 M potassium phosphate buffer (PPB) pH 8. Within a fume hood, we added 5% v/v diethyl ether and 10% v/v chloroform and shook vigorously for 30 s, before centrifuging at max speed (>20,000 × *g*) for 2 min. We then collected the supernatant and passed it through a 0.22 μm nitrocellulose filter to remove bacterial or particulate contaminants. We portioned the filtrate into aliquots, which were later used for RNA extraction or inoculation of pupae after strain identification.

For inocula identification, we obtained RNA from 100 μL of the partitioned filtrate using the RNeasy mini kit (Qiagen). For our sequencing negative control, we extracted RNA from a single juvenile velvet worm (*Euperipatoides rowelli*) using the Direct-zol RNA MiniPrep Plus (Zymo Research). To avoid any potential cross contamination we prepared the velvet worm sample in the Evolutionary and Integrative Zoology Laboratory, University of Sydney. All RNA samples were treated with DNase (Ambion^®^ TURBO DNA-free kit) according to manufacturer’s instructions. We shipped treated RNA (80–150 ng/μL) on dry ice to the Australian Genome Research Facility (AGRF) laboratory (Melbourne, Australia) for preparation of whole transcriptome, 150 bp paired-end libraries with ribosome depletion and MiSeq (Illumina) sequencing.

**FIGURE 1:**
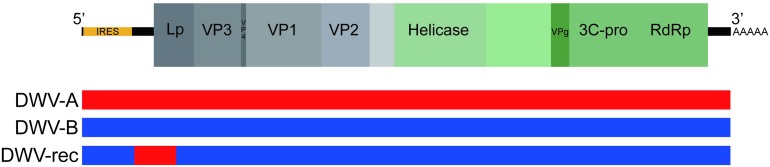
Schematic of the DWV genome structure. Location of coat proteins as per de Miranda and Genersch (2010). DWV-A and DWV-B sequence shown in red and blue, respectively. The Netherlands recombinant (DWV-rec) predominantly corresponds to DWV-B, with a DWV-A region between nucleotide positions 829 and 1487. The first recombination breakpoint occurs after the predicted internal ribosome entry site (IRES), which is predicted to fall within the first 810 nucleotides (Ongus et al., 2006).

Sequencing reads were checked for quality using FastQC^1^ and trimmed to remove residual adaptor sequences and low-quality sequences using Trimmomatic (Bolger et al., 2014). Trimmed reads were assembled *de novo* into contigs using the metagenomic assembler Megahit (Li et al., 2015). Resulting contigs were compared to a custom reference library containing previously identified honeybee virus genome sequences using BLASTn, including but not limited to acute bee paralysis virus (ABPV), *Apis* rhabdovirus (ARV) (Remnant et al., 2017), black queen cell virus (BQCV), chronic bee paralysis virus (CBPV), Israeli acute paralysis virus (IAPV), Kashmir bee virus (KBV), Lake Sinai virus (LSV), and sacbrood virus (SBV). The DWV-A and DWV-rec inocula were negative for all other honeybee viruses, including BQCV. The DWV-B inoculum contained low amounts of LSV and ARV-1 and ARV-2, however, this did not impact our study as we found that these viruses were not transmissible to pupae via injection of our DWV-B inoculum (see section “Results”). In addition, we examined a general viral reference database containing a comprehensive library of viral protein sequences downloaded from GenBank by using BLASTx to identify any potential novel viral sequences. Identified DWV contigs from each source inoculum were aligned to the DWV-A and DWV-B reference genomes in Geneious [Version 10.2.4, (Kearse et al., 2012); accession numbers AJ489744 and AY251269], to produce alternate DWV strain sequence assemblies for each source inoculum. The DWV-A and DWV-B inocula sequences only contained the DWV genotype of interest. The DWV-rec inoculum contained one predominant genotype (Figure 1), where the average coverage per base was approximately 2,800-fold, as estimated by Megahit. Additionally within the DWV-rec inoculum, we detected low frequencies of DWV-B, and an additional recombinant with an extended DWV-A fragment to position 2,140, with low coverage per base values of 26 and 408, respectively (partial contig sequences available as Supplementary Texts S1, S2). The DWV-A, DWV-B and DWV-rec inocula sequences used in this study were deposited to GenBank [accession numbers MN538208–MN538210]. We also compared our inocula sequences to strains previously injected by Gisder et al. (2018), Remnant et al. (2019) and Tehel et al. (2019). We performed pairwise comparisons and nucleotide alignments in Geneious using Muscle, and generated a maximum likelihood phylogenetic tree using PhyML (Supplementary Figure S1 and Supplementary Table S1). We found that our DWV-A and DWV-B inoculum were the most similar to the reference genomes, and more closely related to the inocula used by Remnant et al. (2019) and Tehel et al. (2019), compared to the isolates injected by Gisder et al. (2018).

### Inocula Standardization

#### Quantification of Inocula Viral Load

First strand cDNA was synthesized from DNase treated RNA (0.2–0.4 μg) (Ambion^®^ TURBO DNA-free kit) using SuperScript III Reverse Transcriptase (Invitrogen) with random hexamer primers, in 10 μL reaction volumes. The resulting cDNA was diluted in 30 μL UltraPure nuclease-free dH_2_O (Invitrogen). To determine the viral load of each inoculum we used quantitative PCR (qPCR) to compare quantitation cycle (*C*q) values against the DWV-A and DWV-B standard curves (described below), and multiplied by dilution factor of 1/6,400. We diluted inocula in 0.5 M PPB pH 8.0 to standardize DWV concentration to 1 × 10^7^ genome equivalents (GE). Next, we added 10% green food coloring (Queen, Australia) to visually aid injection.

#### Preparation of DWV-A and DWV-B qPCR Standards

We used absolute quantification with DWV-A and DWV-B plasmid standards to accurately determine viral loads in inocula and injected pupae (Supplementary Figure S2). DWV-A and DWV-B RdRp plasmid standards were prepared from the source material cDNA after strain confirmation (above), using the method adapted from Kevill et al. (2017). We analyzed the cDNA by PCR with the Kapa2G Robust PCR Kit (Kapa Biosystems), as per manufacturer’s instructions, using DWV strain specific RdRp primers (Supplementary Table S2). PCR cycling conditions for all reactions were 94°C (3 min), followed by 35 cycles of 94, 58, and 72°C (30 s), and 72°C (5 min). We analyzed PCR products by gel electrophoresis on 1% agarose gel with SB buffer and SYBR Safe DNA stain (Life Technologies). We cleaned the PCR fragments with GF-1 PCR Clean-up Kit (Vivantis). Plasmid vectors containing DWV-A or DWV-B fragments were prepared with TOPO Cloning reaction and transfected into Transform One Shot TOP10 competent *Escherichia coli* cells (Invitrogen). LB plates with 50 μg/mL kanamycin were prepared as per manufacturer’s instruction, plated with 100 μL cells and incubated at 37°C overnight. We performed colony PCR with DWV strain specific primers to ensure that transformation had occurred, and visualized PCR products on 1% agarose gel (as above). Colonies positive for DWV-A or DWV-B clones were added to 2 mL LB broth with 2 μL kanamycin and incubated at 37°C overnight. We then isolated plasmid DNA with Wizard SV Plus Minipreps DNA Purification System (Promega). As circular plasmids are known to supercoil and produce unreliable absolute qPCR results (Hou et al., 2010), we linearized our plasmids with *PmeI* restriction digest (New England Biolabs). We confirmed linearization on 1% agarose gel, cleaned plasmids (as above), and quantified DNA concentration with Qubit Broad Range Assay. We calculated plasmid copy numbers as per Staroscik (2004), and diluted DWV-A and DWV-B linear plasmids to 5 ng/μL, which was equivalent to 1 × 10^9^ genome copies of DWV.

#### qPCR of DWV Plasmid Standards

We prepared 10-fold serial dilutions from the 1 × 10^9^ plasmid stock to generate DWV-A and DWV-B standard curves from 10^8^ to 10^2^, prior to qPCR analysis with a Bio-Rad CFX384 Touch real-time PCR detection system. We performed all 5 μL qPCR reactions in triplicate with SsoAdvanced Universal SYBR Green supermix (Bio-Rad), forward and reverse primers (final concentration 500 nM each), and 1 μL cDNA, in both DWV-A and DWV-B master mixes. We used the following cycling conditions: 95°C (10 min), followed by 35 cycles of 95°C (30 s), 58°C (30 s) and 72°C (30 s). Melt curve analysis immediately followed between 55 and 95°C, at 0.5°C increments. We plotted average *C*q values against the log_10_ of the plasmid copy number to give a standard curve for DWV-A and DWV-B. PCR primer efficiency (*E* = 10^[–1/slope]^) was 1.91 for DWV-A (slope = −3.5557, *Y*-intercept = 35.165, *R*^2^ = 0.9998) and 1.92 for DWV-B (slope = −3.5343, *Y*-intercept = 35.125, *R*^2^ = 0.9998).

### Pupal Injection Assay and Sample Preparation

#### Experimental Injection of Pupae

We collected approximately 650 white-eyed pupae per colony, from capped brood cells of three unrelated *A. mellifera* colonies kept at the University of Sydney’s apiary. These colonies are naïve to both *V. destructor* and DWV, neither of which are established in Australia (Roberts et al., 2017). Pupae that showed signs of melanization or damage from uncapping were excluded from the assay prior to injection. Mated *V. destructor* females enter a honeybee brood cell just prior to the cell being capped and the bee undergoing pupation (Donzé and Guerin, 1994). The mother mite and her offspring feed on the fat bodies of the developing bee (Ramsey et al., 2019), during which the mother mite can transmit viruses acquired from her previous meal (Bowen-Walker et al., 1999). To mimic the natural vector-mediated infection route as closely as possible, we injected 475 white-eyed pupae per colony; each colony consisted of five treatment groups of 95 pupae. We adapted the injection protocol used by McMahon et al. (2016), to reflect the same viral load and similar DWV treatments. In our study, we injected pupae with either 2 μL of: (1) 0.5 M PPB pH 8 (‘buffer control’), (2–4), 1 × 10^7^ GE of DWV-A, DWV-B, or the recombinant strain (DWV-rec), or (5) an equal mixture (‘co-injection’) containing 5 × 10^6^ GE of DWV-A and DWV-B. In contrast to McMahon et al. (2016), we co-injected pupae with the mixture to assess strain competition during co-infection.

We injected white-eyed pupae with a 32G needle attached to a 10 μL Hamilton syringe inserted between the 3rd and 4th tergites at the side of the abdomen [the typical *V*. *destructor* feeding site (Donzé and Guerin, 1994)], underneath but parallel to the cuticle to avoid puncturing the gut. After injection, we immediately placed pupae into Petri dishes lined with sterile filter paper (10 pupae/Petri dish). We placed the Petri dishes onto shallow racks within clip-locked plastic tubs (Sistema) and incubated at 34.5°C for 8 days (192 h) in the dark. To keep the humidity high, we added 30 mL sterile H_2_O to the plastic tubs housing the Petri dishes.

After injection, we randomly selected four pupae per treatment and colony at regular time-points (1, 4, 8, 12, and 24 h) and every subsequent 24 h for 192 h (just prior to eclosion). Sampled pupae were immediately frozen at −80°C. We continued to incubate the pupae not collected for RNA extraction until 192 h [when remaining pupae were terminated due to Quarantine permit conditions (see below)]. We visually monitored the survival of pupae throughout the experiment, using an adapted version of the method described by Remnant et al. (2019). We used the continual pigment changes in pupal eye and body color (Jay, 1962) as indicators of healthy development. A pupa was classed as dead when eye or body pigments has ceased changing color for 48 h.

#### RNA Extraction and cDNA Synthesis

We extracted RNA from each frozen pupa separately in 1 mL of TRI Reagent (Sigma) with a TissueLyser, according to the manufacturer’s protocol. We suspended RNA pellets in 200 μL ultra-pure water (Invitrogen) and quantified the concentration with Qubit Broad Range Assay (Life Technologies). Samples were standardized to 200 μg/mL RNA to account for body mass differences between individual pupae. First strand cDNA was synthesized from 0.8 μg DNase treated RNA in 10 μL reaction volumes, as described above. The resulting cDNA was diluted in 30 μL UltraPure nuclease-free dH_2_O (Invitrogen).

### qPCR Analysis

#### Viral Analysis of Pupae

For DWV analysis, cDNA from all individual pupae were analyzed in both DWV-A and DWV-B master mixes alongside DWV-A and DWV-B plasmid standards, and positive and negative controls. cDNA from the source inocula was used as positive DWV controls and water served as a negative (no template) control. In addition, we screened all samples for BQCV, SBV and amplified the endogenous control gene, *Actin* (Supplementary Table S2). Whole transcriptome sequencing results indicated that the DWV-B source material was positive for LSV, and ARV-1 and ARV-2. We screened DWV-B injected pupae for ARV-1 and ARV-2 by qPCR, and LSV by endpoint PCR with primers that amplify multiple LSV strains (Supplementary Table S2). The qPCR and 1% agarose gel results showed that these viruses were not transmitted to pupae via injection of DWV-B inoculum.

### Data Analyses

#### Relative Viral Loads

Average *C*q values from triplicate qPCR analyses were confirmed to have a standard deviation of ≤0.3 and we considered *C*q values ≥ 35 to be DWV free. A small number of samples (3.2% of 720 pupae) randomly distributed across each treatment and colony had abnormal amplification of DWV or *Actin*. These cases included two pupae which had very high average *C*q values of 31.2 and 28.3 for *Actin*, three pupae with abnormally low DWV loads for their time-point, and 17 pupae where DWV-A or DWV-B was detected in the opposing qPCR master mix to what was injected. We excluded these pupae from further analyses and attributed these anomalies to possible pupal death, error during injection, and contamination during downstream processing, respectively. Though we suspected it unlikely, we wanted to ensure that the three pupae with low DWV loads for their time point (one pupa injected with DWV-rec from Colony 2 at 192 h post-injection, and two co-injected pupae from Colony 3 at 144 h post-injection) were not true reflections of natural variation between individual pupae. Thus, we repeated the statistical analyses with these three individuals included (see section “Results” for further details).

We measured the accumulation of viral loads in pupae from 8 to 192 h post-injection, relative to housekeeping gene *Actin* to account for any precision error during preparation and handling of samples. Primer efficiencies were calculated using the slope of the standard curve constructed with a 10-fold dilution series of cDNA, from 10^0^ to 10^–6^ (Supplementary Table S2). We determined the relative loads of DWV strains or BQCV with the Pfaffl expression ratio (Pfaffl, 2001), which mathematically corrects for differences in primer efficiencies. The calculation compares the primer efficiency (*E*) and *C*q difference (Δ) of the target virus (DWV strain or BQCV) to those of reference gene *Actin*, in individual pupae versus buffer controls. We assigned buffer injected pupae a *C*q of 40 for their viral value, as they were negative for DWV and BQCV.

#### Absolute DWV Viral Loads

Absolute viral loads in DWV injected pupae were interpolated from mean *C*q values against the associated standard curve and multiplied by dilution factor (9/4000). This gave the absolute viral load as DWV genome equivalents in cDNA synthesized from 0.8 μg RNA. Mean absolute viral loads per treatment and colony (8–192 h post-injection) have been provided in the Supplementary Material (Supplementary Figure S2) so that our results can be compared to other studies.

### Statistical Analyses

#### Accumulation and Competition

To determine if there were significant differences in mean viral loads (relative to *Actin*) between genotypes over time we used a two-way ANOVA followed by Tukey (HSD) *post hoc* analysis. As viral loads rapidly increased over many orders of magnitude within the first 48 h (exponential phase of replication) and were visibly different between genotypes, we chose to analyze the most linear and consistent phase of the data, from 48 to 192 h post injection. A visual assessment of the homogeneity of variance (Residual vs. Fitted plot) and normality (Normal QQ plot) assumptions showed that a fourth root transformation of the response variable (mean DWV load) substantially improved the model. We used backward elimination based on Akaike’s Information Criterion (AIC) values to fit the most parsimonious model. All statistical analyses were performed with RStudio software (R version 3.5.0).

#### Survival

We analyzed the survival of pupae throughout the incubation period when exposed to the five different injection treatments. At each specific time-point pupae were assigned a survival value of 0 if alive or censored (removed for viral analysis), or 1 if dead. As the data did not meet the Cox proportional hazards assumption, we analyzed the mean proportion of pupal survival with a generalized linear mixed effects model (glmer) with binomial distribution and logit link function (“lme4” package) (Bates et al., 2018). Again, the most parsimonious model was determined with backward elimination based on AIC values. We then analyzed the final model as a type II ANOVA (“car” package) (Fox et al., 2018), followed by Tukey pairwise comparison analysis using lsmeans function (“lsmeans” package) (Lenth, 2018).

### Quarantine Permit

Frozen adult honeybees (workers) containing Deformed wing virus were imported from New Zealand and the Netherlands under our Department of Agriculture and Water Resources import permit 0000917783. The permit allows us to infect local honeybee pupae with DWV within our strictly controlled Quarantine approved laboratory at the University of Sydney; however, all pupae must be terminated prior to eclosion. Thus, the remaining pupae that were not collected at earlier time points for viral analysis were terminated at 192 h post-injection, prior to eclosion.

## Results

### Viral Accumulation Post-injection

**FIGURE 2:**
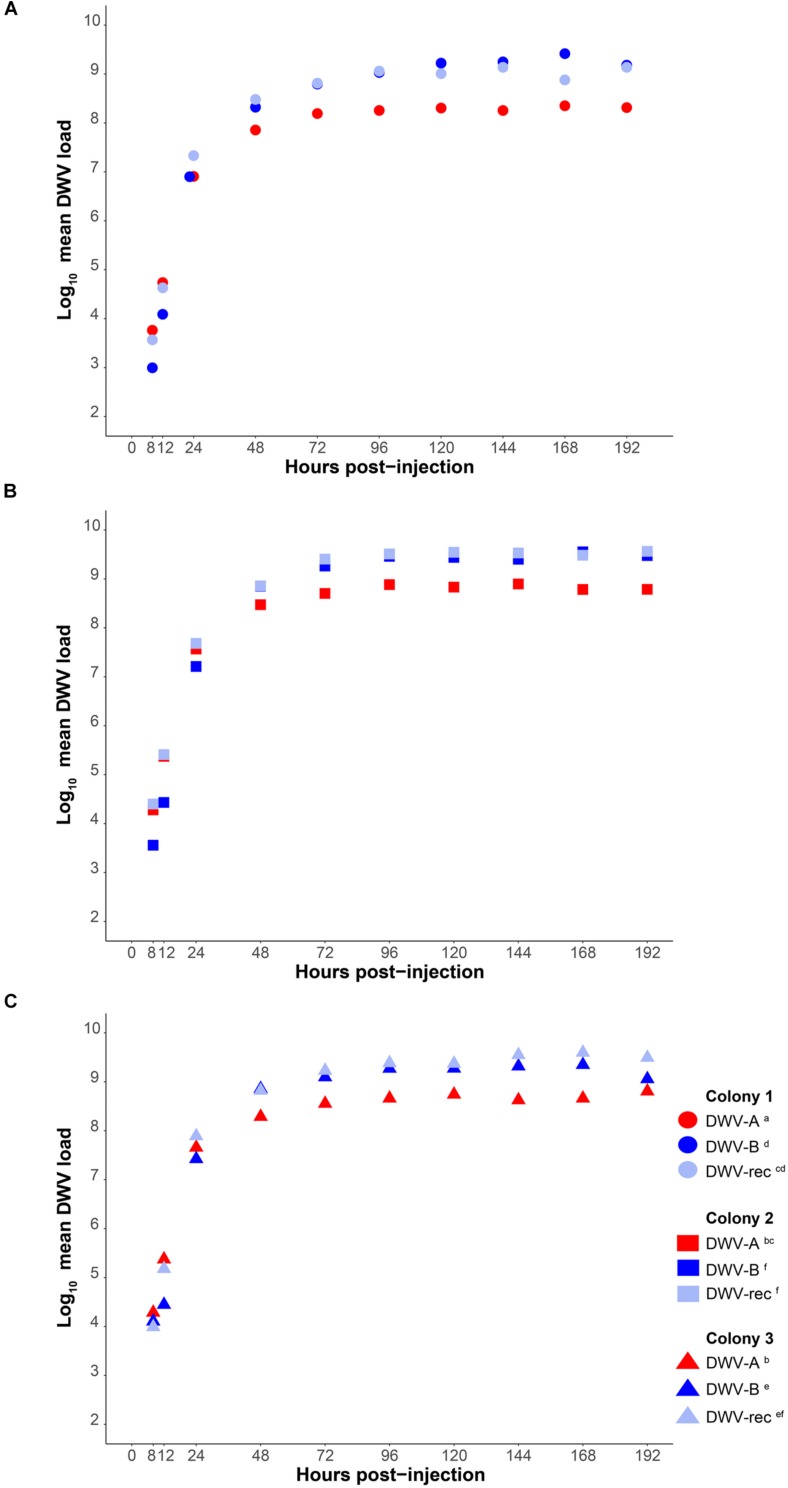
Mean DWV viral loads of individually infected pupae from 8 to 192 h post-injection (*n* = 120 per treatment), relative to housekeeping gene *Actin* in cDNA synthesized from 0.8 μg RNA. White-eyed pupae from three naïve colonies **(A–C)** were singly injected with 1 × 10^7^ genome equivalents of DWV-A, DWV-B or recombinant strain (‘DWV-rec’). Viral loads rapidly increased over several orders of magnitude within the first 48 h of infection (exponential replication phase). Statistical analyses were performed in the linear phase of the data, from 48 to 192 h post-injection. Significant (*p* < 0.05) differences between genotypes and colonies indicated with lettering. See the electronic Supplementary Material, Supplementary Tables S3, S4 for details of the statistical analyses.

**FIGURE 3:**
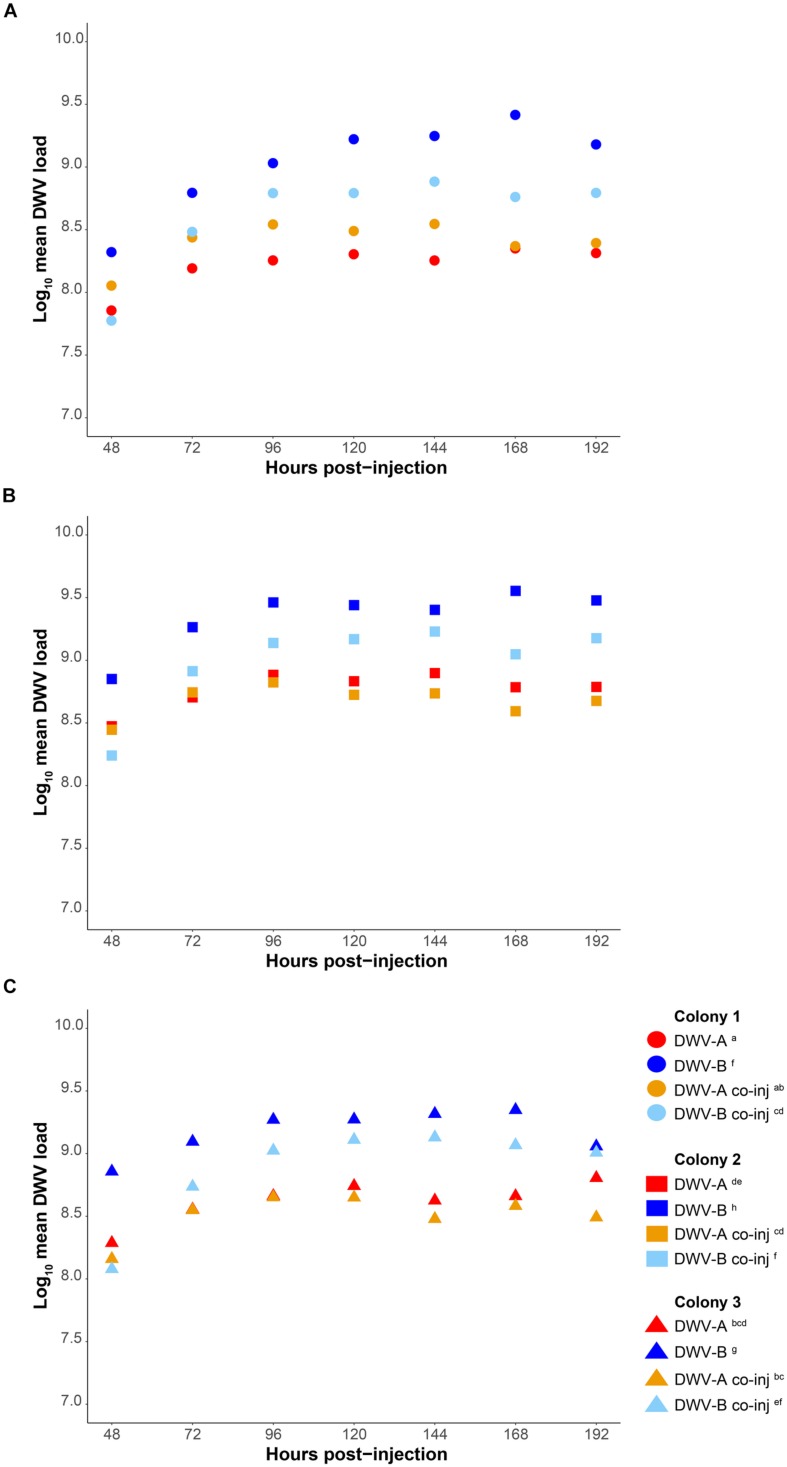
Comparison of mean DWV viral loads of individual pupae singly infected with DWV-A or DWV-B versus pupae co-injected with 5 × 10^6^ genome equivalents of DWV-A and DWV-B, from 48 to 192 h post-injection. Viral loads are relative to housekeeping gene *Actin* in cDNA synthesized from 0.8 μg RNA. Accumulation loads are displayed by colony **(A–C)**. Significant (*p* < 0.05) differences between genotypes and colonies indicated with lettering. Viral loads of singly injected pupae also shown in Figure 2.

We measured the accumulation of viral loads relative to housekeeping gene *Actin* and as absolute genome copy equivalents by standard curve (Supplementary Figure S2) in individual pupae using qPCR with cDNA synthesized from 0.8 μg RNA. Our method does not measure any level of viral degradation by the honeybee immune response and therefore reflects the net virus levels, assuming that a combination of viral replication and degradation occurs. DWV was not detected in any of our buffer injected pupae (*n* = 144). The average *C*q values for DWV in pupae at 1 and 4 h post-injection were >30, inconsistent between samples, and in some individuals DWV was not detected at all. Thus, these time points were excluded from further analyses. DWV was detected in all DWV-injected pupae from 8 h post-injection onwards. Viral loads of all genotypes (DWV-A, DWV-B, and DWV-rec) rapidly increased within the first 48 h post-injection, either when injected alone (Figure 2 and Supplementary Figure S3) or co-injected (DWV-A and DWV-B) (Figure 3 and Supplementary Figure S4), and plateaued between 72 and 96 h post-injection. We found that accumulation patterns were more dynamic within the first 48 h, with high variation between genotypes and colonies. Despite this variation, DWV-B loads were generally lower than DWV-A and DWV-rec, particularly in colonies 1 and 2. However, DWV-B loads in all colonies exceeded DWV-A from 72 h post-injection. This remained true when DWV-A and DWV-B were co-injected excluding DWV-B loads in colony 1 compared to DWV-A loads in colonies 2 and 3 (Figure 3).

To determine if there was a significant difference in the net accumulation of viral loads from 48 to 192 h post-injection, we compared the mean viral loads of all genotypes relative to *Actin* (injected singly or co-injected) with an ANOVA followed by Tukey (HSD) *post hoc* analysis. We found significant differences in mean viral loads amongst all genotypes (*F* = 248.642; *df* = 4, 381; *p* < 0.0001), and found that the strains differed in the rate at which they accumulated, as indicated by a significant strain × time interaction (*F* = 15.325; *df* = 4, 381; *p* < 0.0001) (Supplementary Table S3). DWV-B accumulated to significantly higher loads compared to DWV-A in all colonies [Tukey (HSD) *post hoc p* < 0.0001; Figure 2 and Supplementary Table S4], and viral loads were 5- to 10-fold higher than DWV-A. The accumulation of DWV-B and DWV-rec were not significantly different within the same colonies (*p* < 0.05). However, mean viral loads were significantly affected by colony (*F* = 147.876; *df* = 2, 381; *p* < 0.0001), although the overall pattern of increase over time remained the same. We found that colony 1 pupae injected with DWV-A or DWV-rec had significantly lower loads compared to colonies 2 and 3 (*p* < 0.0001), and that DWV-rec loads in colony 1 were not significantly different to DWV-A loads in colony 3 (*p* = 0.1358). Similarly, DWV-B loads were significantly different between all colonies (*p* < 0.05). We also found a significant interaction between strain and colony (*F* = 4.837; *df* = 8, 381; *p* < 0.0001). Upon reanalysis, the inclusion of the three pupae with low loads for their time point did not alter the significance of any of the predictor variables (Supplementary Table S5). However, we did find slight differences in the pairwise comparisons of colonies; DWV-B loads between colonies 1 and 3, and 2 and 3 were no longer significantly different (*p* > 0.05) (Supplementary Table S6).

We classified competition between DWV genotypes as a significant reduction in mean viral loads of DWV-A or DWV-B when co-injected compared to singly from 48 to 192 h post-injection, relative to *Actin*. Co-injected DWV-B loads were significantly lower than when DWV-B was injected alone in all colonies (*p* < 0.0001; Figure 3 and Supplementary Table S4). In contrast, DWV-A loads did not differ when injected singly or when co-injected (*p* > 0.05). As when injected singly, we found that DWV-A loads in co-injected pupae were significantly lower in colony 1 compared to colony 2, and DWV-B loads were significantly lower in colony 1 compared to colonies 2 and 3 (*p* < 0.05). We found slight differences in these results with the inclusion of the three additional pupae, whereby DWV-A loads in co-injected pupae were significantly higher (*p* < 0.05) in colony 2 compared to both colony 1 and 3 (Supplementary Table S6).

### Survival

**FIGURE 4:**
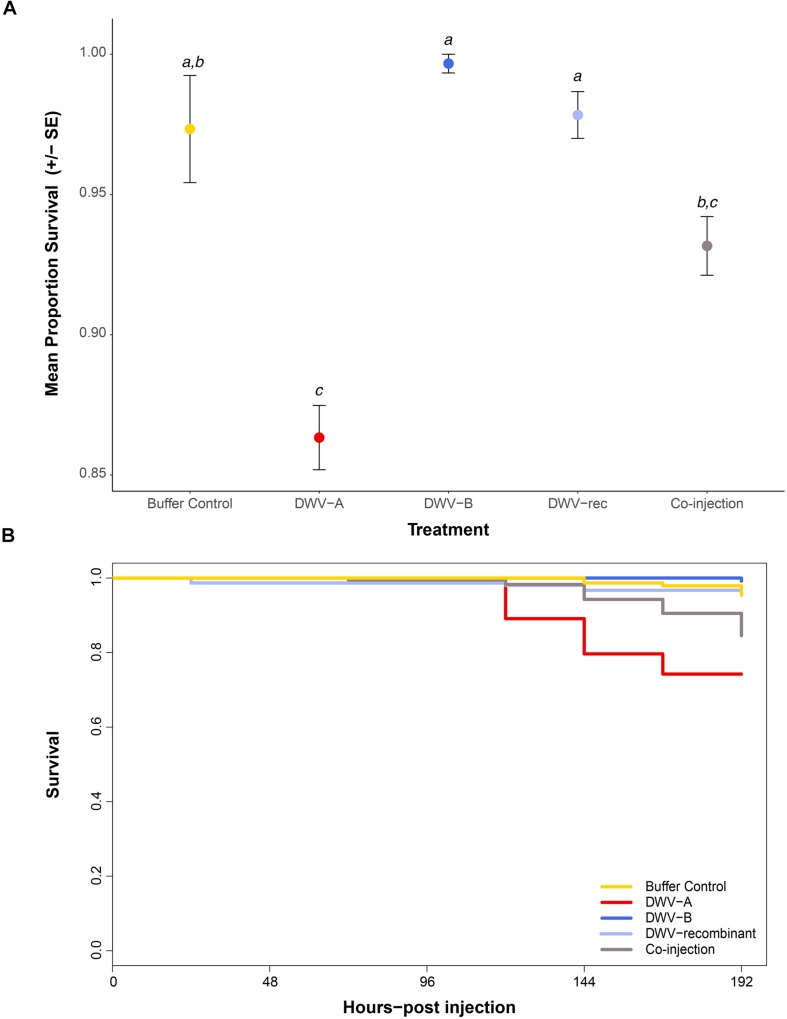
Survival of individual naïve pupae singly injected with DWV-A, DWV-B, DWV recombinant (‘DWV-rec’), buffer control, or co-injected with DWV-A and DWV-B (*n* = 285 per treatment). Pupal survival was monitored throughout the incubation period, up to 192 h post-injection. Pupae collected at regular time points for viral analysis were recorded as censored, thus the final number of remaining pupae at 192 h post-injection was *n* ≤ 141 per treatment (depending on mortality). **(A)** Mean proportion and standard error of pupal survival at 192 h post-injection. Letters show significant differences between treatments (*p* < 0.05) based on pairwise comparisons of the final model. See the electronic Supplementary Material, Supplementary Tables S7–S9 for details of statistical analyses. **(B)** Survival curve of pupae by treatment up to 192 h post-injection. Data did not meet the Cox proportional hazards assumption, thus **(B)** only used to illustrate the pattern of mortality over time.

We monitored the survival of pupae throughout the incubation period when exposed to the five different injection treatments. At each specific time-point pupae were assigned a survival value of 0 if alive or censored (removed for viral analysis), or 1 if dead. While the vast majority of pupae survived the injections, survival was significantly affected by treatment (χ^2^ = 44.472; *df* = 4; *p* ≤ 0.0001) (Supplementary Table S7). The survival of pupae singly or co-injected with DWV-A did not significantly differ (*p* = 0.1095; Figure 4A and Supplementary Table S8). However, only pupae singly injected with DWV-A had mortality that significantly differed from the buffer control (*p* ≤ 0.0001), and only after 120 h post-injection (Figure 4B). The survival of pupae injected with DWV-B or DWV-rec did not significantly differ from that of the buffer controls (*p* > 0.05). ‘Colony’ had no effect on survival (using Akaike’s information criterion during backward elimination; Supplementary Table S9).

### Presence of Other Viruses in Injected Pupae

**FIGURE 5:**
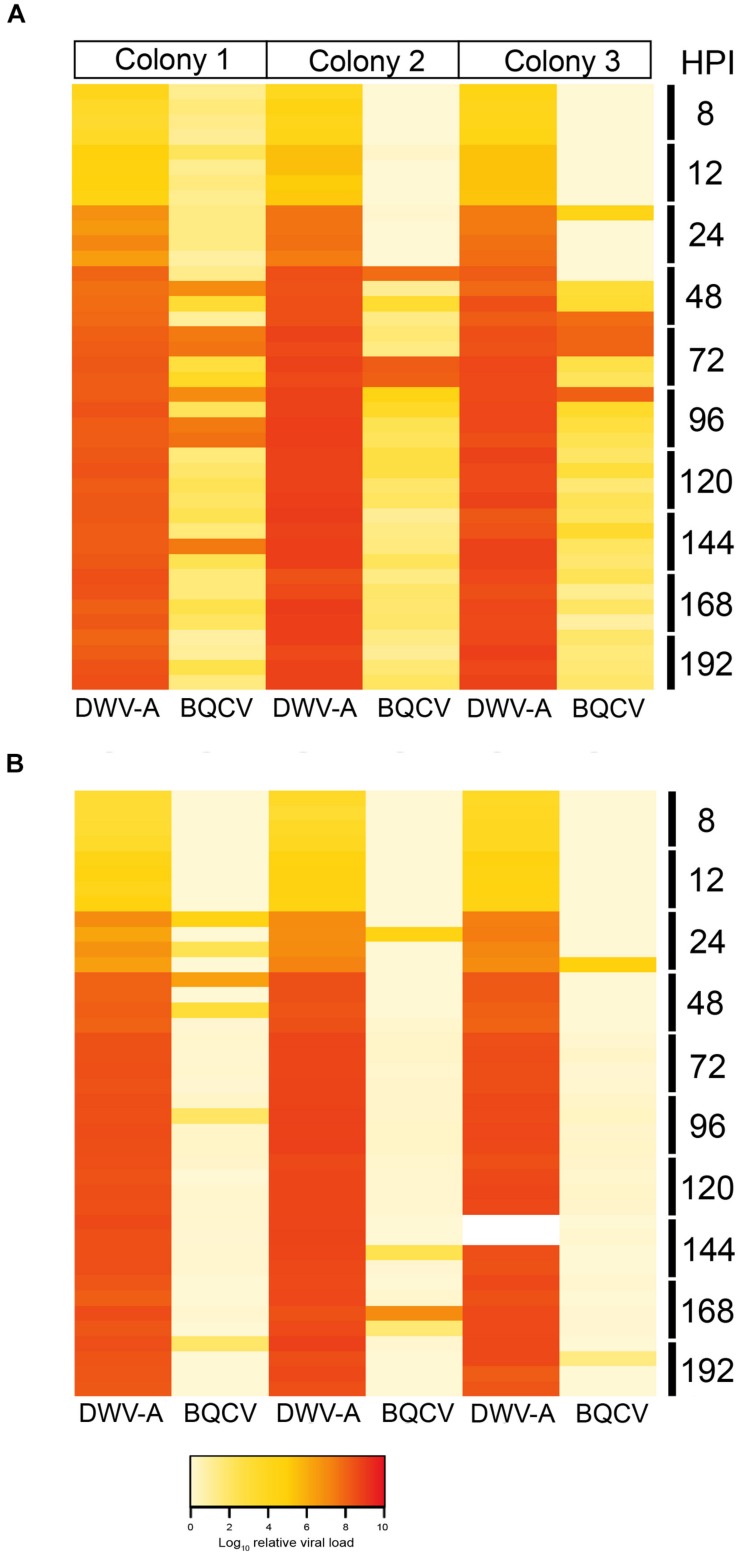
Heat map showing the log_10_ relative viral loads of DWV-A in comparison to BQCV per pupa and colony, in single DWV-A **(A)** and co-injected pupae **(B)**, from 8 to 192 h post-injection. The two white cells in colony 3 (co-injected DWV-A at 144 h post-injection) had abnormally low DWV loads for their time-point, thus were excluded from analyses.

Because our DWV-B inoculum contained LSV, and ARV-1 and ARV-2 we screened the DWV-B injected pupae for LSV by endpoint PCR with primers designed to amplify multiple variant strains, and ARV-1 and ARV-2 by qPCR (Supplementary Table S2). LSV, ARV-1 and ARV-2 were not detected in any of the DWV-B injected pupae, suggesting that these viruses were not transmissible via injection of the DWV-B inoculum. The DWV-A and DWV-rec inocula were negative for all other known honeybee viruses. However, we additionally chose to screen all injected pupae for BQCV and SBV via qPCR as Remnant et al. (2019) previously found covert infections of both viruses in our honeybee population. We did not detect SBV in any pupae across all colonies. In contrast, we detected BQCV in some of the DWV-A and co-injected pupae, yet BQCV was not detected in any of the DWV-B, DWV-rec or buffer injected pupae across the three colonies. The relative BQCV loads varied highly between individuals and colonies (Figure 5). BQCV had higher prevalence in pupae singly injected with DWV-A, where the virus accumulated to loads ≥ 1 × 10^7^ in 4.9% of pupae, predominantly between 48 and 96 h post-injection (Figure 5). Less than 1% of co-injected pupae had BQCV loads of 10^7^ or more. Unlike the DWV strains, BQCV loads did not continuously increase over time. Peak BQCV loads coincided with the commencement of mortality observed with DWV-A injections, at 120 h post-injection (Figure 4B).

## Discussion

Our aim was to assess the ability of three genotypes of DWV (DWV-A, DWV-B, and DWV-rec) to accumulate in honeybee pupae naïve to DWV and *V. destructor*, both in isolation and during co-infection. Our experimental protocol resulted in a rapid infection with all DWV genotypes accumulating to viral loads exceeding 1 × 10^7^ (relative to housekeeping gene *Actin* or 1 × 10^9^ genome copy equivalents by standard curve) within 48 h. Viral loads typically plateaued at 96 h post-infection, in agreement with previous analysis by ELISA (Martin et al., 2013). We found significant differences in the relative mean viral loads of the two master variants, which differed by an order of magnitude in colony 1, and approximately fivefold in colonies 2 and 3. While DWV-A loads initially accumulated faster, DWV-B ultimately reached significantly higher levels from 72 h post-injection. Our results are consistent with the experimental findings of McMahon et al. (2016); Dubois et al. (2019), and Tehel et al. (2019) who also found that DWV-B accumulates to higher loads than DWV-A when injected into pupae or adults, despite all four studies using different honeybee populations. While McMahon et al. (2016) and Tehel et al. (2019) used the same source of inocula, the inocula used by Dubois et al. (2019) and our study were different. Given the consistency in results despite the differences amongst the four studies, we can safely conclude that DWV-B reaches higher viral loads than DWV-A after injection. Similarly to our study, in English and Welsh colonies (Kevill et al., 2019) and in the United States (Ryabov et al., 2017; Kevill et al., 2019), mean DWV-B loads were approximately sevenfold higher than DWV-A when colonies contain both genotypes. Yet there are exceptions. In some co-infected colonies in the United States that died over winter, Kevill et al. (2019) found significantly higher DWV-A loads relative to DWV-B.

Only DWV-B appears to be affected by competition when co-injected. We found that DWV-B loads were significantly reduced in pupae co-injected with DWV-A across all colonies. Interestingly by 96 h post-infection, DWV-B still accumulated to higher loads than DWV-A during co-infection, excluding colony 1. DWV-A loads, when co-injected, were not significantly different to single DWV-A injections, despite containing half the starting dose. Thus, competition appears to be independent of the initial dose. Our results are in accordance with Tehel et al. (2019) even though Tehel et al. (2019) injected a much lower dose (10^2^–10^4^ genome equivalents) and quantified virus levels in far fewer samples (*n* = 4–11). It thus seems there is a maximum level that DWV-A can accumulate to. This is in agreement with Ryabov et al. (2019), who found that five divergent DWV-A clones all accumulated to the same level. While we observed evidence of competition between DWV-A and DWV-B, we did not see strong competitive exclusion between the genotypes, suggesting that the reduction in DWV-B loads was not due to a lack of some critical resource required for viral replication. In contrast, Israeli acute paralysis virus (IAPV) and closely related Kashmir bee virus (KBV) appear to compete directly for cellular resources. In the presence of KBV, accumulation of IAPV was reduced by four orders of magnitude (Carrillo-Tripp et al., 2016). Lastly, we found significant differences in both single and co-injected DWV loads between colonies, indicating that colony-level factors, such as immune response (Niu et al., 2014; Brutscher et al., 2015), might additionally affect DWV accumulation.

Our recombinant strain (DWV-rec) accumulated to equally high loads as DWV-B within the same colonies. Previous studies have shown that some recombinant strains can replicate to higher loads than the master variants (Moore et al., 2011; Zioni et al., 2011; Ryabov et al., 2014). The genome structure of DWV-rec predominantly corresponds to DWV-B, with two recombination breakpoints at positions 829 and 1487 (when aligned to DWV-B AY251269), resulting in a DWV-A region from the 5′ untranslated region (UTR) up to approximately the first half of the Leader protein (Lp). This structure differs from previously characterized recombinants, which have a breakpoint within the helicase region and subsequent non-structural proteins corresponding to DWV-A (Moore et al., 2011; Zioni et al., 2011; Ryabov et al., 2014; Dalmon et al., 2017). Interestingly, the 5′ end of our DWV-rec strain is similar to the RecVT-Fr1 strain isolated from a *V. destructor* tolerant colony in France (Dalmon et al., 2017), although our breakpoints occur earlier.

The difference in accumulation amongst the genotypes may potentially be due to the way viruses interact with cellular translational machinery. Many RNA viruses, including *Dicistroviridae*, *Flaviviridae*, and *Picornaviridae*, use internal ribosome entry site (IRES) secondary structures to initiate translation of their open read frame(s) (Martínez-Salas, 2008). The predicted IRES of DWV-A and DWV-B fall approximately within the first 810 nucleotides (Ongus et al., 2006), prior to our first breakpoint at position 829 for DWV-rec. Thus, the IRES of DWV-rec corresponds to DWV-B. DWV-A and DWV-B share approximately 84% nucleotide and 95% amino acid homology (Ongus et al., 2004). While their 5′ UTR sequences differ, the overall IRES structures were predicted to be the same (Ongus et al., 2006). Nevertheless, small sequence differences may result in different translational efficiencies. For example, a single nucleotide mutation (C472U) in the IRES reduces poliovirus type 3 replication and virulence in mouse neural tissue (La Monica et al., 1987), but does not affect organ tropism (Kauder and Racaniello, 2004). At this stage, it is unclear exactly what part of the genome is most important for DWV replication. However, it is possible that the increased accumulation of DWV-B and DWV-rec compared to DWV-A might be associated with sequence differences within the IRES.

We found differences in mortality between pupae injected with different genotypes. In agreement with Dubois et al. (2019) and Tehel et al. (2019), we found no relationship between viral accumulation and mortality in pupae. In our study, only pupae singly injected with DWV-A showed mortality statistically different from the buffer control, but mortality was low (11% ± 1.2 SE). Interestingly, the 0.4% mortality we observed in DWV-B injected pupae up to 192 h (8 days) post-injection was less than the 55%, 0-75%, and 18% mortality observed by Gisder et al. (2018), Dubois et al. (2019), and Tehel et al. (2019), respectively, after 7 or 10 days post-injection.

Gisder et al. (2018) postulate that their high pupal mortality is further evidence that DWV-B is more virulent than DWV-A, yet the independent DWV-B isolates injected in our study and by Tehel et al. (2019) were found to be more similar (99.3 and 98.9% pairwise identity, respectively) to the DWV-B reference genome [AY251269; isolated from *V*. *destructor* by Ongus et al. (2004)] than the three isolates injected by Gisder et al. (2018) (91.4–96.9%). Furthermore, the DWV-_P0_ I isolate injected by Gisder et al. (2018) shows recombination with DWV-A, however, Gisder et al. (2018) did not indicate whether mortality differed between their three DWV-_P0_ isolates. Dubois et al. (2019) associated their high mortality with SBV, initially present at very low levels in their inocula. While Dubois et al. (2019) and Tehel et al. (2019) found no difference in mortality between DWV-A and DWV-B injected pupae, this may be affected by background DWV infection in their pupae. Both studies detected accumulation of both DWV genotypes upon injection of a single genotype, accumulation of both genotypes in buffer injected pupae and had higher control mortality (11–25%) than observed in our study (2.7% ± 1.9 SE).

While we did find significant mortality when pupae were injected with DWV-A, we caution that we cannot exclude that the mortality was attributed to BQCV and not DWV-A, particularly as BQCV is known to kill brood (Chen and Siede, 2007). Unfortunately, we were unable to screen our 48-h dead pupae for viruses due to extreme RNA degradation, thus cannot determine if dead pupae were infected with BQCV. Nevertheless, we only detected significant mortality in pupae injected with DWV-A, and only detected BQCV in pupae injected with the DWV-A genotype. As BQCV was not detected in any of our inocula by whole transcriptome sequencing, it seems unlikely that we injected BQCV together with DWV. While we cannot completely exclude the possibility that BQCV was present in our DWV-A inoculum at levels too low to be detected, we think this unlikely because we did detect low amounts of LSV and ARV in the DWV-B inoculum. It could be that DWV-A has an immunosuppressive effect that then allows other viruses, such as BQCV, to replicate to high viral loads as suggested by Barroso-Arévalo et al. (2019a). In a 21 months study of honeybee colonies in Spain, Barroso-Arévalo et al. (2019a) found that BQCV, in addition to DWV and *V*. *destructor*, was highly prevalent and negatively correlated with colony vigor. As our study was conducted in the absence of *V*. *destructor*, our results may point to a synergistic interaction between DWV-A and BQCV, such that injection with DWV-A activates an endogenous BQCV infection, potentially by disrupting immune response of pupae more than other DWV genotypes. D’Alvise et al. (2019) also suggested a potential synergistic interaction between DWV and BQCV. Their regression analysis showed that DWV was the most significant predictor of BQCV accumulation in German honeybees, despite contrasting seasonal dynamics and BQCV being significantly correlated to virtually all of the tested viral pathogens and intestinal parasites (D’Alvise et al., 2019). In agreement with Barroso-Arévalo et al. (2019a), D’Alvise et al. (2019) postulated this interaction may be associated with a reduction in host immune defense by DWV. As DWV-A and DWV-B were combined for analysis (D’Alvise et al., 2019), it is unclear if their results would differ between DWV genotypes. We found no evidence of a relationship between DWV-B and BQCV, or between our DWV-rec strain and BQCV. Previous modeling has shown that 20% pupal mortality associated with *Varroa* transmission of DWV to pupae can lead to colony mortality, due to a reduction in workforce longevity (Martin, 2001). While the mortality observed for DWV-A in our study was less than this, any increased effect of DWV-A on the mortality of pupae, with or without an interaction with BQCV, can explain the shift from DWV-A to DWV-B observed globally. Because the reproductive success of *V*. *destructor* depends on the pupa surviving to adulthood, DWV-A associated pupal mortality will negatively affect the transmission of DWV-A in favor of the transmission of DWV-B.

Our data provide some explanation for the continued global increase in prevalence of DWV-B over DWV-A. Low mortality in pupae and the ability of DWV-B to accumulate to higher loads relative to DWV-A, even during co-infection, are likely to be contributing factors to the increasing prevalence of DWV-B. Further, our observed interaction between DWV-A and BQCV highlights the complex relationships between viruses. Previous studies have suggested, implicitly and explicitly, that studying a single virus in isolation does not provide the whole picture (Mondet et al., 2014; Carrillo-Tripp et al., 2016; Dubois et al., 2019; Remnant et al., 2019), particularly as honeybees are frequently infected with multiple pathogens (Bailey et al., 1981; Chen et al., 2004; Berényi et al., 2006; Berthoud et al., 2010; Nguyen et al., 2011; Cornman et al., 2012; Locke et al., 2014; Mondet et al., 2014; Amiri et al., 2015; Natsopoulou et al., 2017; D’Alvise et al., 2019). While a direct relationship between DWV-A and BQCV requires experimental validation, our results suggest that future studies should continue to incorporate a broader ecological approach by experimentally investigating how multiple pathogens interact with their honeybee hosts.

## Data Availability Statement

The inocula sequences used in this study were deposited to GenBank [accession numbers MN538208-MN538210].

## Author Contributions

AN, ER, and MB conceived the study. AN conducted the experimental work. AN and GB carried out molecular laboratory work. AN analyzed the data. AN, ER, and MB contributed to data interpretation and writing the manuscript. All authors gave final approval for publication.

## Conflict of Interest

The authors declare that the research was conducted in the absence of any commercial or financial relationships that could be construed as a potential conflict of interest.

## Footnotes

1 http://www.bioinformatics.babraham.ac.uk/projects/fastqc/
