# Supplementary material for: Accumulation and Competition Amongst Deformed Wing Virus Genotypes in Naïve Australian Honeybees Provides Insight Into the Increasing Global Prevalence of Genotype B
Source: Front Microbiol. 2020 Apr 9;11:620. doi: 10.3389/fmicb.2020.00620 (PMC7160646; doi:10.3389/fmicb.2020.00620)
Supplement: Supplementary file 1 [file Data_Sheet_1.pdf]

Supplementary Material

Accumulation and Competition Amongst Deformed Wing Virus Genotypes in Naïve Australian Honeybees Provides Insight Into the Increasing Global Prevalence of Genotype B

Amanda M Norton, Emily J Remnant, Gabriele Buchmann, and Madeleine Beekman

This file contains

- Supplementary Figure S1-S4
- Supplementary Tables S1-S9
- Supplementary Text S1-S2
- Supplementary References

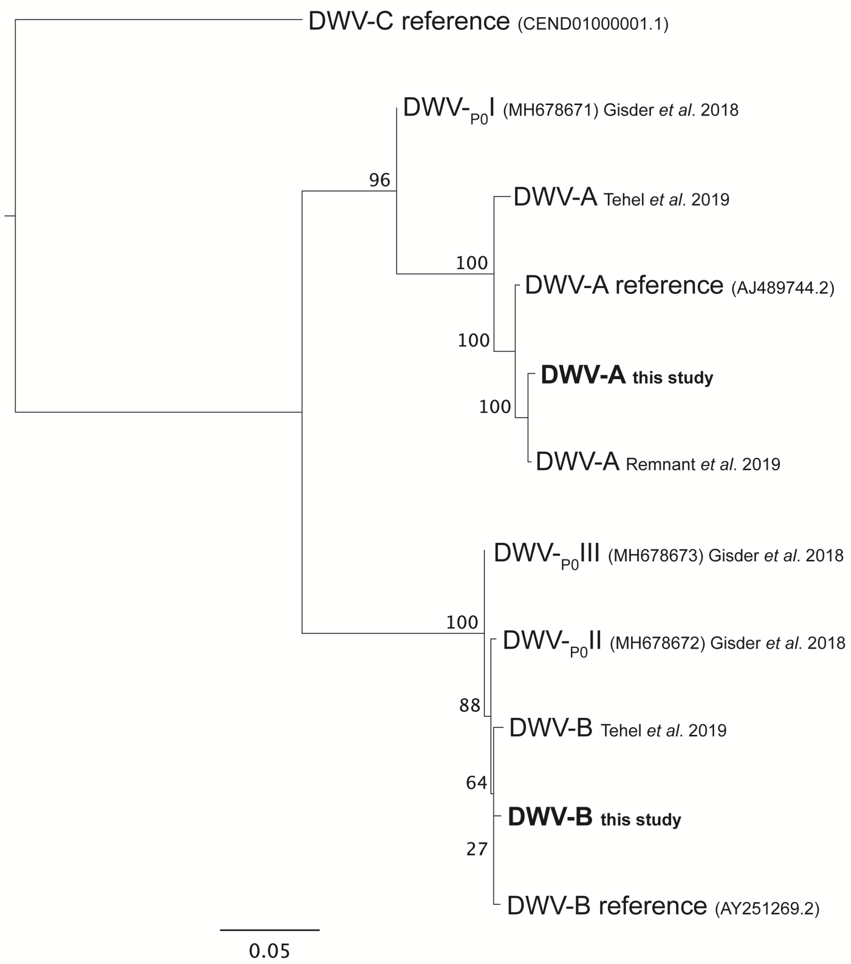

Figure S1. Phylogenetic tree comparing whole genome sequences of the DWV-A (accession number MN538208) and DWV-B (MN538209) inocula obtained in this study to isolates experimentally injected by Remnant et al. (2019), Tehel et al. (2019) and Gisder et al. (2018) (MH678671-73). DWV-A (AJ489744.2; Lanzi et al. (2006)), DWV-B (AY251269.2; Ongus et al. (2004)) and DWV-C (CEND01000001; (Mordecai et al., 2016)) were used as reference sequences, with DWV-C as the outgroup. The DWV-A and DWV-B whole genome sequences from this study were obtained from transcriptome sequencing (see Materials and Methods) and are highlighted in bold. Sequences were aligned in Geneious using Muscle and trimmed where required. Maximum likelihood trees were produced using PhyML HKY85 model after 100 bootstrap replicates.

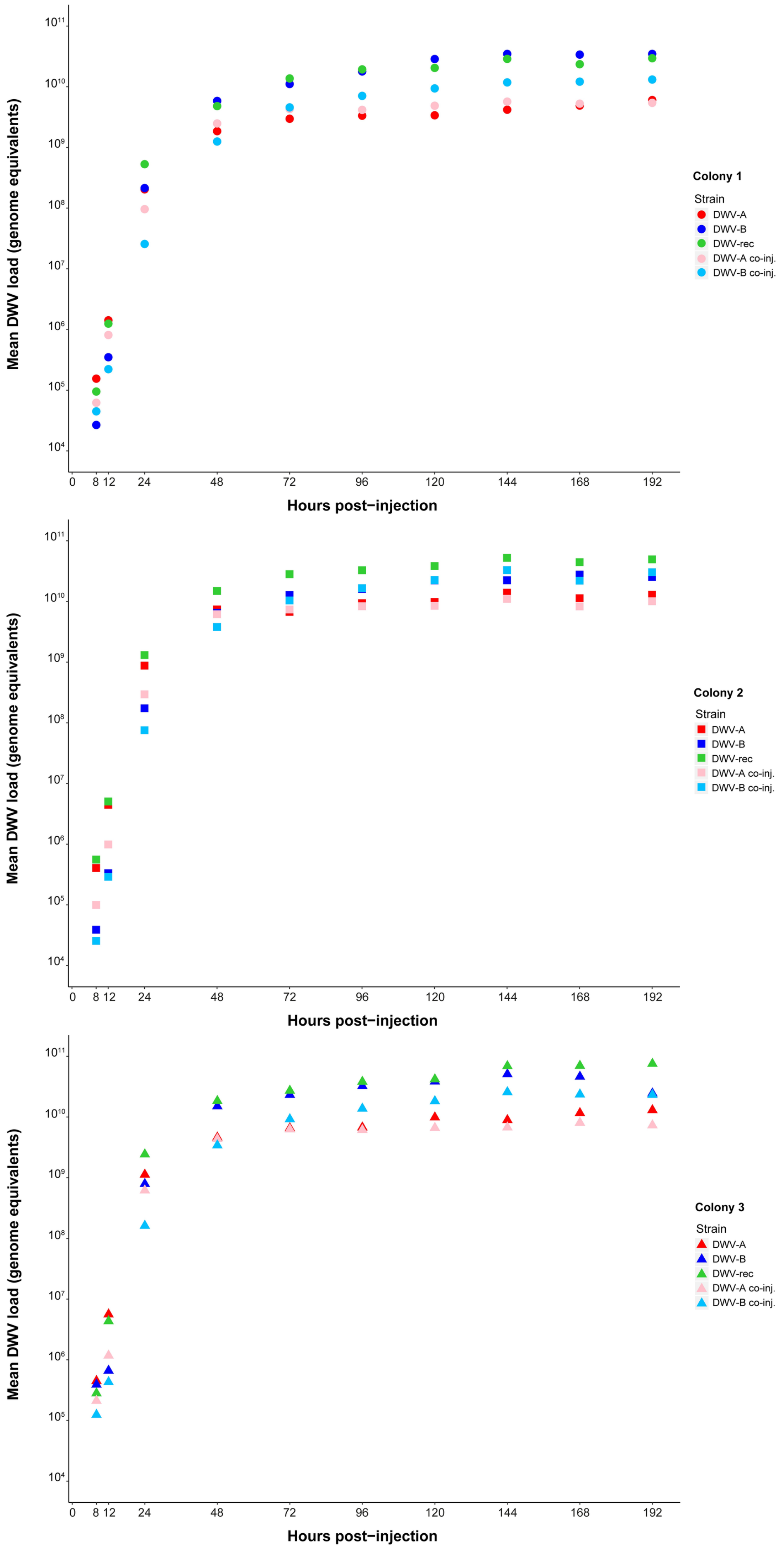

Mean DWV load (genome equivalents)
10^{4} 10^{5} 10^{6} 10^{7} 10^{8} 10^{9} 10^{10} 10^{11}
0 8 12 24 48 72 96 120 144 168 192
Hours post−injection
Colony 1
Strain
DWV-A
DWV-B
DWV-rec
DWV-A co-inj.
DWV-B co-inj.
Mean DWV load (genome equivalents)
10^{4} 10^{5} 10^{6} 10^{7} 10^{8} 10^{9} 10^{10} 10^{11}
0 8 12 24 48 72 96 120 144 168 192
Hours post−injection
Colony 2
Strain
DWV-A
DWV-B
DWV-rec
DWV-A co-inj.
DWV-B co-inj.
Mean DWV load (genome equivalents)
10^{4} 10^{5} 10^{6} 10^{7} 10^{8} 10^{9} 10^{10} 10^{11}
0 8 12 24 48 72 96 120 144 168 192
Hours post−injection
Colony 3
Strain
DWV-A
DWV-B
DWV-rec
DWV-A co-inj.
DWV-B co-inj.

Figure S2. Mean DWV viral loads (genome equivalents) of individually infected pupae from 8 to 192 hours post-injection (n = 120 per treatment), as calculated by standard curve in cDNA synthesized from 0.8 µg RNA. White-eyed pupae from colonies one to three were singly injected with 1 × 10^7 genome equivalents of DWV-A, DWV-B or recombinant strain ('DWV-rec'), or co-injected with 5 × 10^6 genome equivalents of DWV-A and DWV-B. Each figure displays the mean viral load by colony to illustrate colony differences.

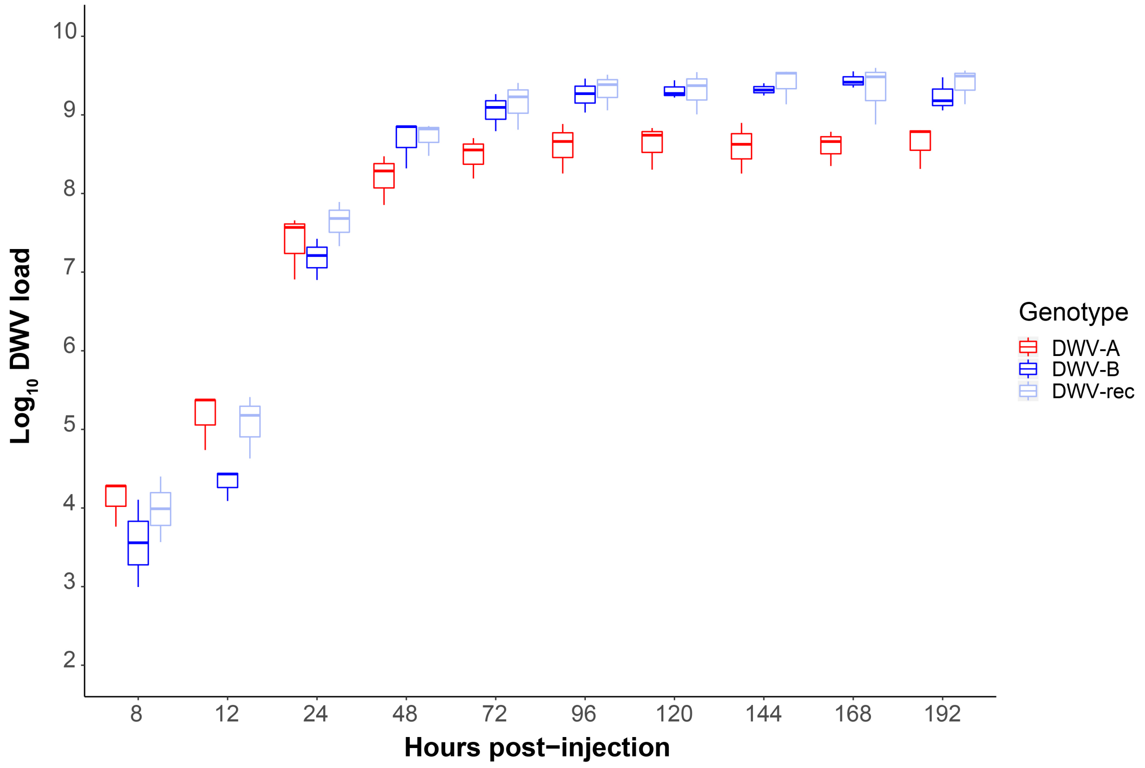

Figure S3. Log_{10} DWV viral loads of singly infected pupae from 8 to 192 hours post-injection, relative to housekeeping gene *Actin*. Colonies 1-3 have been combined (per treatment) to illustrate the distribution of viral accumulation per genotype.

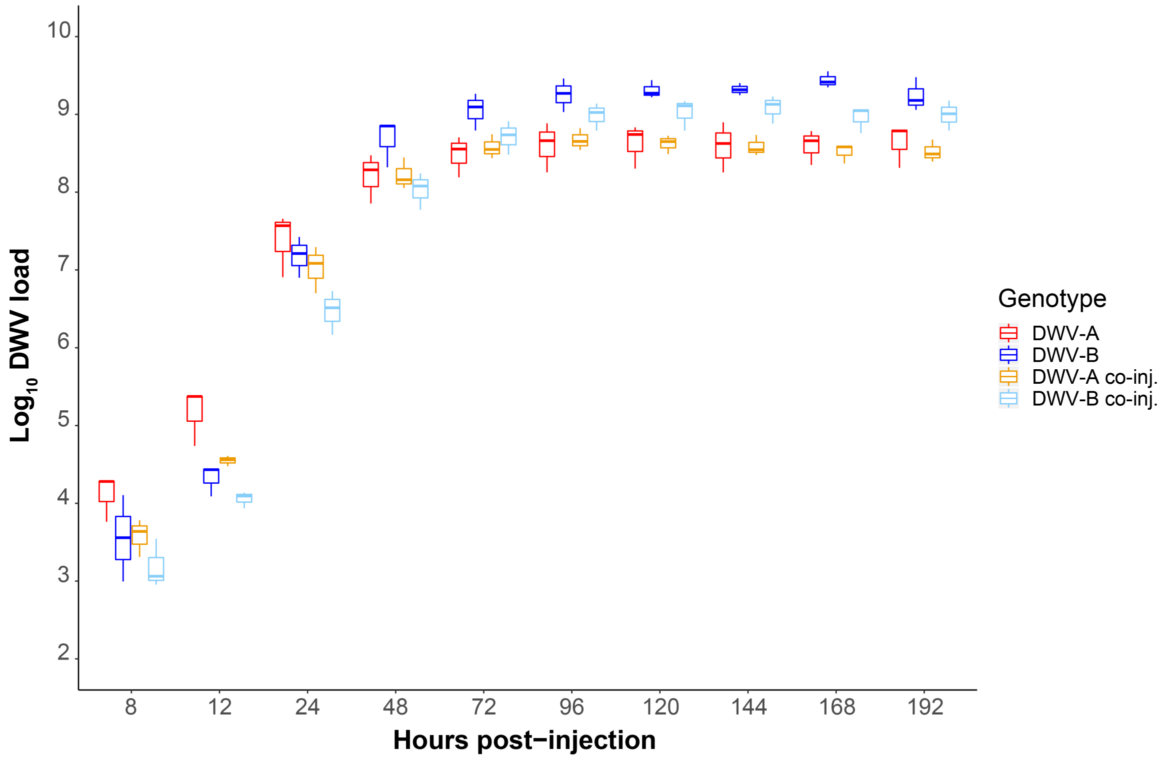

Figure S4. Comparison of $\log_{10}$ DWV viral loads of pupae singly infected with DWV-A or DWV-B versus pupae co-injected with DWV-A and DWV-B, relative to housekeeping gene *Actin*. Colonies 1-3 have been combined (per treatment) to illustrate the distribution of viral accumulation per genotype.

Table S1. Comparison of pairwise identity values, generated from pairwise Muscle alignments of whole genome sequences in Geneious. DWV-A (MN538208) and DWV-B (MN538209) inocula sequences obtained in this study were compared to DWV-A (AJ489744.2) and DWV-B (AY251269.2) reference sequences, in addition to the DWV-A isolate we previously injected in Remnant et al. (Remnant et al., 2019) and those experimentally injected by Tehel et al. (2019) and Gisder et al. (2018).

	DWV-A (this study) MN538208	DWV-A (AJ489744.2) (Lanzi et al., 2006)	DWV-A Remnant et al. (2019)	DWV-A Tehel et al. (2019)	DWV-$_{P0}$ I Gisder et al. (2018)	DWV-$_{P0}$ II Gisder et al. (2018)
DWV-A (AJ489744.2)	98.50%					
DWV-A Remnant et al. (2019)	99.50%	98.72%				
DWV-A Tehel et al. (2019)	97.30%	97.70%	97.46%			
DWV-$_{P0}$ I Gisder et al. (2018)	91.20%	91.50%	91.29%	91.80%		
DWV-$_{P0}$ II Gisder et al. (2018)	84.70%	85.00%	84.87%	84.90%	90.70%	
DWV-$_{P0}$ III Gisder et al. (2018)	85.80%	85.90%	85.85%	86.00%	91.60%	94.50%

	DWV-B (this study) MN538209	DWV-B (AY251269.2) (Ongus et al., 2004)	DWV-B Tehel et al. (2019)	DWV-$_{P0}$ I Gisder et al. (2018)	DWV-$_{P0}$ II Gisder et al. (2018)
DWV- B (AY251269.2)	99.30%				
DWV-B Tehel et al. (2019)	99.20%	98.90%			
DWV-$_{P0}$ I Gisder et al. (2018)	91.40%	91.40%	91.40%		
DWV-$_{P0}$ II Gisder et al. (2018)	95.00%	94.90%	94.90%	90.70%	
DWV-$_{P0}$ III Gisder et al. (2018)	96.90%	96.80%	96.80%	91.60%	94.50%

Table S2. List of the primers used for viral detection by endpoint PCR and quantitative PCR. (ARV: *Apis* rhabdovirus, BQCV: black queen cell virus, DWV: deformed wing virus, LSV: Lake Sinai virus, SBV: sacbrood virus).

Virus	Primer name	Sequence	Product size (bp)	Primer efficiency	Reference
**LSV**	LSV_2108_F	TCATCCMAAGAGAACCA	400	-	This study
	LSV_uni_R	GTCAAAGGTGTCGTATCC			
**qPCR primers**					
**DWV**	DWV_F	TACTAGTGCTGGTTTTCCTTT	155	1.91	Kevill et al. (2017)
	DWV-A_R	CTCATTAACTGTGTCGTTGAT			
	DWV-B_R	CTCATTAACTGAGTTGTTGTC	155	1.92	
**BQCV**	BQCV_qPCR_F	AGGTTTACGCTCCAAGATCG	112	1.73	Remnant et al. (2019)
	BQCV_qPCR_R	TTTGTTCAGCAGGTAAATTGTTC			
**SBV**	SBV_ qPCR_F	CGAGGAGGGAAAAACTACGC	115	-	Remnant et al. (2019)
	SBV_ qPCR_R	GTGGCTTAACTGGATCATAGCC			
**ARV-1**	ARV-1_F	ATGAGGCTTGGAGACACAGC	100	-	This study
	ARV-1_R	GGAGCTTTCCTGAGGACACG			
**ARV-2**	ARV-2_F	CTAAACCCCACCTGTCTGCC	150	-	This study
	ARV-2_R	ATTGAGCACTGGAGCGTTGG			
Actin	Actin_F	TGCCAACACTGTCCTTTCTG	155	1.70	Scharlaken et al. (2008)
	Actin_R	AGAATTGACCCACCAATCCA			

Table S3. Two-way ANOVA comparing mean DWV loads relative to housekeeping gene *Actin* in pupae singly injected with DWV-A, DWV-B, DWV-rec, or co-injected with DWV-A and DWV-B, from 48 to 192 hours post injection (n = 401). Comparison of DWV loads between genotype and colonies (1-3) over time. Data plotted in Figure 2 and Figure 3, where we have presented the mean relative viral loads as single injection by colony (DWV-A, DWV-B and DWV-rec), and single injection versus co-injection (DWV-A and DWV-B), respectively.

**Summary**	***df***	**Sum sq**	**Mean sq**	**F value**	***p***
DWV genotype	4	344421	86105	248.642	<0.0001
hour post injection (HPI)	1	76077	76077	219.685	<0.0001
colony	2	102420	51210	147.876	<0.0001
DWV genotype:HPI	4	21228	5307	15.325	<0.0001
DWV genotype:colony	8	13400	1675	4.837	<0.0001
residuals	381	131941	346		

Table S4. Tukey HSD post-hoc analysis of two-way ANOVA (S3 Table), showing pairwise comparisons of mean relative viral loads by genotype and colony. Lettering denotes genotype followed by numbering 1-3, which indicates colony (A: DWV-A single injection, B: DWV-B single injection, R: DWV-rec single injection, CA: DWV-A co-injection, and CB: DWV-B co-injection). Significant comparisons ($p < 0.05$) denoted as letters on Figure 2 and Figure 3.

**Comparison**	***df***	**t.ratio**	***p***	**Comparison**	***df***	**t.ratio**	***p***
A,1 - B,1	381	-13.138	<.0001	R,1 - A,2	381	3.061	0.1358
A,1 - CA,1	381	-2.643	0.3451	R,1 - B,2	381	-9.501	<.0001
A,1 - CB,1	381	-6.152	<0.0001	R,1 - CA,2	381	4.412	0.0013
A,1 - R,1	381	-11.214	<.0001	R,1 - CB,2	381	-1.563	0.9654
A,1 - A,2	381	-8.205	<0.0001	R,1 - R,2	381	-9.900	<.0001
A,1 - B,2	381	-21.109	<.0001	R,1 - A,3	381	5.429	<.0001
A,1 - CA,2	381	-6.931	<.0001	R,1 - B,3	381	-5.388	<.0001
A,1 - CB,2	381	-13.02	<.0001	R,1 - CA,3	381	6.912	<.0001
A,1 - R,2	381	-22.646	<.0001	R,1 - CB,3	381	0.546	1.0000
A,1 - A,3	381	-5.679	<.0001	R,1 - R,3	381	-9.075	<.0001
A,1 - CA,3	381	-16.008	<.0001	R,2 - A,3	381	16.592	<.0001
A,1 - CA,3	381	-4.175	0.0033	R,2 - B,3	381	5.018	<.0001
A,1 - CB,3	381	-10.871	<.0001	R,2 - CA,3	381	18.093	<.0001
A,1 - R,3	381	-20.457	<.0001	R,2 - CB,3	381	11.874	<.0001
A,2 - B,2	381	-12.71	<.0001	R,2 - R,3	381	1.955	0.0631
A,2 - CA,2	381	1.337	0.9916	CA,1 - CB,1	381	-3.509	0.0371
A,2 - CB,2	381	-4.696	<.0004	CA,1 - R,1	381	-8.62	<.0001
A,2 - R,2	381	-14.311	<.0001	CA,1 - A,2	381	-5.585	<.0001
A,2 - A,3	381	2.418	0.5027	CA,1 - B,2	381	-18.467	<.0001
A,2 - B,3	381	-8.31	<.0001	CA,1 - CA,2	381	-4.289	0.0021
A,2 - CA,3	381	3.915	0.0091	CA,1 - CB,2	381	-10.377	<.0001
A,2 - CB,3	381	-2.567	0.3956	CA,1 - R,2	381	-20.028	<.0001
A,2 - R,3	381	-12.221	<.0001	CA,1 - A,3	381	-3.089	0.1263
A,3 - B,3	381	-10.497	<.0001	CA,1 - B,3	381	-13.572	<.0001
A,3 - CA,3	381	1.481	0.9783	CA,1 - CA,3	381	-1.582	0.9617
A,3 - CB,3	381	-4.983	<.0001	CA,1 - CB,3	381	-8.229	<.0001
A,3 - R,3	381	-14.502	<.0001	CA,1 - R,3	381	-17.863	<.0001
B,1 - CA,1	381	10.545	<.0001	CA,2 - CB,2	381	-6.089	<.0001
B,1 - CB,1	381	7.102	<.0001	CA,2 - R,2	381	-15.779	<.0001
B,1 - R,1	381	1.891	0.8565	CA,2 - A,3	381	1.118	0.9987
B,1 - A,2	381	4.969	<.0001	CA,2 - B,3	381	-9.62	<.0001
B,1 - B,2	381	-7.576	<.0001	CA,2 - CA,3	381	2.626	0.3557
B,1 - CA,2	381	6.337	<.0001	CA,2 - CB,3	381	-3.94	<.0083
B,1 - CB,2	381	0.363	1.000	CA,2 - R,3	381	-13.655	<.0001
B,1 - R,2	381	-9.206	<.0001	CA,3 - CB,3	381	-6.493	<.0001
B,1 - A,3	381	7.319	<.0001	CA,3 - R,3	381	-15.989	<.0001
B,1 - B,3	381	-3.602	<.0001	CB,1 - R,1	381	-5.177	<.0001

B,1 - CA,3	381	8.802	<.0001	CB,1 - A,2	381	-2.109	0.7284
B,1 - CB,3	381	2.471	0.4639	CB,1 - B,2	381	-14.958	<.0001
B,1 - R,3	381	-7.184	<.0001	CB,1 - CA,2	381	-0.779	1.000
B,2 - CA,2	381	14.178	<.0001	CB,1 - CB,2	381	-6.869	<.0001
B,2 - CB,2	381	8.089	<.0001	CB,1 - R,2	381	-16.551	<.0001
B,2 - R,2	381	-1.730	0.9227	CB,1 - A,3	381	0.353	1.000
B,2 - A,3	381	15.026	<.0001	CB,1 - B,3	381	-10.338	<.0001
B,2 - B,3	381	3.445	0.0454	CB,1 - CA,3	381	1.862	0.8704
B,2 - CA,3	381	16.54	<.0001	CB,1 - CB,3	381	-4.720	<.0003
B,2 - CB,3	381	10.238	<.0001	CB,1 - R,3	381	-14.42	<.0001
B,2 - R,3	381	0.259	1.000	CB,2 - R,2	381	-9.745	<.0001
B,3 - A,1	381	-16.008	<.0001	CB,2 - A,3	381	7.091	<.0001
B,3 - CA,3	381	11.894	<.0001	CB,2 - B,3	381	-4.008	<.0064
B,3 - CB,3	381	5.988	<.0001	CB,2 - CA,3	381	8.602	<.0001
B,3 - R,3	381	-3.154	0.1062	CB,2 - CB,3	381	2.149	0.7009
				CB,2 - R,3	381	-7.680	<.0001
				CB,3 - R,3	381	-9.788	<.0001

Table S5. Two-way ANOVA repeated as above, with the inclusion of three pupae with low DWV loads for their time-point.

**Summary**	***df***	**Sum sq**	**Mean sq**	**F value**	***p***
DWV strain	4	351858	87965	167.09	<0.0001
hour post injection (HPI)	1	65820	65820	125.03	<0.0001
colony	2	94129	47064	89.40	<0.0001
DWV strain:HPI	4	21683	5421	10.20	<0.0001
DWV strain:colony	8	16258	2032	3.86	<0.001
residuals	384	202685	526		

Table S6. Tukey HSD post-hoc analysis of repeated two-way ANOVA (Table S5), showing the three pairwise comparisons that were significantly different in the repeated analysis.

**Comparison**	***df***	**t.ratio**	***p***
B,1 - B,3	384	-3.038	0.144
B,2 - B,3	384	2.74	0.2848
CA,2 - CA,3	384	3.592	0.0282

Table S7. Analysis of the mean proportion of survival of pupae subjected to five treatments (buffer control, DWV-A, DWV-B, DWV-rec and co-injection) at 192 hours post injection. We fit a generalized linear mixed effects model (glmer) with binomial distribution and logit link function, comparing mean proportion of survival per treatment. Colony had no effect on survival (using Akaike's information criterion during backward elimination; Table S9), thus was included in the model as a random factor. We then analysed the final model as a type II ANOVA. As treatment was a significant predictor of survival, we subsequently ran a Tukey pairwise comparison (Table S8).

**Response: Proportion Survival**	**Chi sq**	***df***	***p***
(Intercept)	92.583	1	< 0.0001
treatment	44.472	4	< 0.0001

Table S8. Tukey post-hoc analysis of ANOVA (Table S7); pairwise comparisons of mean proportion of survival between treatments.

**Comparison**	**z. ratio**	***p***
Buffer Control - DWV-A	-4.384	< 0.0001
Buffer Control - DWV-B	1.835	0.3532
Buffer Control - DWV-rec	0.280	0.9987
Buffer Control - Co-injection	-2.586	0.0728
DWV-A - DWV-B	3.747	0.0017
DWV-A - DWV-rec	4.467	< 0.0001
DWV-A - Co-injection	2.422	0.1095
DWV-B - DWV-rec	-1.671	0.4522
DWV-B - Co-injection	-3.036	0.0203
DWV-rec - Co-injection	-2.778	0.0435

Table S9. Model testing of mean proportion of survival data per treatment (buffer control, DWV-A, DWV-B, DWV-rec and co-injection) and colony. Akaike's information criterion obtained during backward elimination showed that colony was not a significant predictor of survival. Colony was thus included as a random factor in survival analyses.

	df	**Deviance**	**AIC**	**LRT**	***p***
		512.07	526.07		
treatment	4	579.34	585.34	67.275	< 0.0001
colony	2	514.71	524.71	2.641	0.267

Text S1. Partial contig sequence of the DWV-B isolate detected in low frequency in the DWV-rec inoculum, where Megahit average coverage per base was 408.0453.

>DWV-Rec_Netherlands_k141_295_multi=408.0453
AATCATATATTACTTTCTTAATTTTAAATAATAACATGGGCGATCGATTACAAACGGGCCTAACAACTTTCTCGCGCACAAAT
TTTGATGCCACCCGTTTCACTTTCTTACAAATCTTTGTATATTTGGGTTTCTTAAATACGAACTCACCCGCGTCTTTTCCTACCT
CAATCTTAAGGGATTCTAAAGGGGAATATCTATTACTGATTGAAATGGGGACATGTTCTTGAACAGGGCGGCGGGTACATCT
TTCAGCTATAGAATATATCGTGTATAAATTATTCAATTGTTCCGTAAGGAACTCATTATCACGCGCATCTTCTTGCTCCCACGT
TGTATGATCATACACAGTGACGTCAAGAACGTTTCGAATCCGTTCCTGTTCCAACGCCAAACGCTTAATAACGCGTCGACGC
CTAGCTTCATCAATCTCCCAACTACGGGGAGCATGAGCTACAGAGGGAGCTTGGGCAACAGCAGCATAAGAAAGAGTTCCA
CAACTAAATGCCATTTTTATATATATTTACCTTCAAAGTCAAAATCAAATTGAAAATATAAAATAAAAAGAATTGAATCTAA
TATATAAAGCAAAAATAGCAAAGAAATATATTTAGCAATAATGAAAATAGCAAAATATAATAGCAAATAAAAACAGCAAAA
TAAAACAGCAATAATCGTAGCATTAAAATATTAAATAATGTCTTTCCATTCGTCATCGTCTGGGCTATGTGACATTAACGGGT
GCCCTGCGAACGTAACGTACCGTGGCATAAATTATACTGTATTAAAAGGATTCTTGCCAAAGGAAATCCCAATACAGTCACT
TATGTATAGAGACATAAATTACTTTCACACTTTCGCCTCATACA

Text S2. Partial contig sequence of the secondary DWV-recombinant isolate detected at very low frequency in the DWV-rec inoculum, where Megahit average coverage per base was 26.3092.

>DWV-Rec_Netherlands_k141_7331_multi=26.3092
AAGAATGGATATACGTGTTTAAAAGGTATCACTAATTTTGCTTCATTACTCGCTGATGCGCTAATCAAAGCATGATCCATATG
CGAAAAACCATACACACTTCGCTTCGTCTGGATATTCAAATTTTCATGATCCGAATAATACCAAGTTGCCTGTAATTGACCAA
CTTGGAATTTATTTGAATTAATTTGAACTCTAACTTCCATATCGCCTCGCCAATATGCGTGCACCTTAAATGGGATAGTATTA
GGCACATCACATATAGCATCAGAATTAGCCTCTATACTAGATAACAAAGCACGAGGCAAAATTAAACGTGCTAACTCCTTAT
CAAATGGATCATCCTTCGACCAAACAAATTCAGCAATCTGATACCATCGAGATGTAATTGTGGCATAATCATCTACTACATC
ATTACTAGTCCATCTACTCCATTTTACGCTGACCGGCGCTGGAATAGATGTACTAGGATCTCGTTGAGTCGTTAAAACAACAT
TACTATCCTTTTCTAATTCAACTTCACCCTCGCCATCAGGTCCTGGATTAGGGTTATCCATCTCTGGTTTTGCCTGCACCGGAT
TCGATAATTGTAACAGACTAGTGACACACTCTAACTCATAATCGCGCTGTTTTTGACGTCGCAACATCCTAATCTGTTTTCTTA
ATCTATATAAATGCAAATCATAAATTATCTTCTTAAGCTTAAATAATAGCATAGGGGATCTAGAACATATAGGACGAACAAC
TTTTTCACGAACGAAGCGAGTTGCAACGCGCTTCACTTTCTTGCAAACGCGCGTATATTTAGGTTTCTTAAATATACATTCGC
CTGCTTCTTGACCGACCTCGACCTTAAGGGATTCCAGTGGAGCAAACCTATTCGAAACTGATATAGGAGAGTACTCTTTGATA
GGCCGACGCGTACAACGTTCAGCGATCGAATAAATAGTATATAAGTTGTTTAATTGTTCCATTAGGAACTCATTATCGCGCGC
GTCCTCCTGTTCCCACGTTGCCTGGTCATAGACGTCAGCGTCAAGCACGTTACGAATACGTTCTTGCTCCAACGCCAAACGTT
TAATAACTCGGCGCCGCCTAGCTTCATCTACTTCCCATGTACGAGGTGCATGGGCGACAGATGGAGCTTGAGCGACGGCAGA
GTAAGAAAGAATTCCACAACTAAATGCCATTTTTATATATATTTACCTTGAAAATCAAAATCAAATTGAAAATATAAAATAA
AAAGAATTGAATCTAATATATAAAGCAAAAATAGCAAAGAAATATATTTAGCAATAATGAAAATAGCAAAATATAATAGCA
AATAAAAACAGCAAAATAAAACAGCAATAA

References

Gisder, S., Möckel, N., Eisenhardt, D., and Genersch, E. (2018). In vivo evolution of viral virulence: switching of deformed wing virus between hosts results in virulence changes and sequence shifts. *Environmental Microbiology* 20(12), 4612–4628. doi: 10.1111/1462-2920.14481.

Kevill, L.J., Highfield, A., Mordecai, J.G., Martin, J.S., and Schroeder, C.D. (2017). ABC Assay: Method Development and Application to Quantify the Role of Three DWV Master Variants in Overwinter Colony Losses of European Honey Bees. *Viruses* 9(11), 314. doi: 10.3390/v9110314.

Lanzi, G., de Miranda, J.R., Boniotti, M.B., Cameron, C.E., Lavazza, A., Capucci, L., et al. (2006). Molecular and biological characterization of deformed wing virus of honeybees (*Apis mellifera* L.). *Journal of virology* 80(10), 4998-5009. doi: 10.1128/JVI.80.10.4998–5009.2006.

Mordecai, G.J., Wilfert, L., Martin, S.J., Jones, I.M., and Schroeder, D.C. (2016). Diversity in a honey bee pathogen: first report of a third master variant of the Deformed Wing Virus quasispecies. *The ISME journal* 10(5), 1264-1273. doi: 10.1038/ismej.2015.178.

Ongus, J.R., Peters, D., Bonmatin, J.M., Bengsch, E., Vlak, J.M., and van Oers, M.M. (2004). Complete sequence of a picorna-like virus of the genus *Iflavirus* replicating in the mite *Varroa destructor*. *Journal of General Virology* 85(12), 3747-3755. doi: 10.1099/vir.0.80470-0.

Remnant, E.J., Mather, N., Gillard, T., Yagound, B., and Beekman, M. (2019). Direct transmission by injection affects competition amongst RNA viruses in honeybees. *Proceedings of the Royal Society B: Biological Sciences* 286(1895), 20182452. doi: 10.1098/rspb.2018.2452.

Scharlaken, B., De Graaf, D.C., Goossens, K., Brunain, M., Peelman, L.J., and Jacobs, F.J. (2008). Reference gene selection for insect expression studies using quantitative real-time PCR: The head of the honeybee, *Apis mellifera*, after a bacterial challenge. *Journal of Insect Science* 8(33), 1-10. doi: 10.1673/031.008.3301.

Tehel, A., Vu, Q., Bigot, D., Gogol-Döring, A., Koch, P., Jenkins, C., et al. (2019). The Two Prevalent Genotypes of an Emerging Infectious Disease, Deformed Wing Virus, Cause Equally Low Pupal Mortality and Equally High Wing Deformities in Host Honey Bees. *Viruses* 11(2), 114. doi: 10.3390/v11020114.
